# Mild Abiotic Stress Affects Development and Stimulates Hormesis of Hemp Aphid *Phorodon cannabis*

**DOI:** 10.3390/insects12050420

**Published:** 2021-05-08

**Authors:** Roma Durak, Malgorzata Jedryczka, Beata Czajka, Jan Dampc, Katarzyna Wielgusz, Beata Borowiak-Sobkowiak

**Affiliations:** 1 Department of Experimental Biology and Chemistry, University of Rzeszów, Pigonia 1, 35-310 Rzeszów, Poland; rdurak@univ.rzeszow.pl (R.D.); jdampc@ur.edu.pl (J.D.); 2 Institute of Plant Genetics, Polish Academy of Sciences, Strzeszynska 34, 60-479 Poznań, Poland; mjed@igr.poznan.pl; 3 Department of Entomology and Environmental Protection, Poznan University of Life Sciences, Dąbrowskiego 159, 60-594 Poznań, Poland; beataczajka1996@gmail.com; 4 Department of Breeding and Agronomy of Fibrous and Energy Plants, Institute of Natural Fibers and Medicinal Plants–National Research Institute, Wojska Polskiego 71B, 60-630 Poznań, Poland; katarzyna.wielgusz@iwnirz.pl

**Keywords:** temperature, herbicide, enzymatic markers, *Cannabis sativa*, demographic parameters

## Abstract

**Simple Summary:**

For centuries, hemp has been used by humans as a source of natural fiber. Nowadays, it is a multipurpose crop with many uses, but the most valuable are hemp metabolites including terpenes and cannabinoids. During the cultivation of hemp, farmers encounter wilting plants, inhabited by the hemp aphid—a persistent plant-damaging insect. The species lives mainly on the undersides of leaves and on flower stalks and feeds on the phloem sap. We studied the effects of temperature and the herbicide used by farmers in routine hemp cultivation on the population biology of hemp aphids. Hemp aphids thrived best at moderate temperatures between 20 and 25 °C. At this temperature range, they lived for about 25 days, during which they reproduced for 15 days, producing between 54.5 and 111.6 nymphs in total per female. At 28 °C, aphid survival and reproductive capacity were much lower. Treatment of plants with herbicide caused mild stress in aphids and resulted in increased aphid reproduction and a change in their behavior; aphids settled on lower parts of the plant rather than on the top part of the plant (growing point), which is normal in untreated plants. This new knowledge may help to manage the hemp aphid and reduce damage to hemp crops in the future.

**Abstract:**

The hemp aphid *Phorodon cannabis* Passerini is a well- known (Asia, Europe) or newly emerging (North America) insect. It is a monophagous insect pest causing considerable damage in field and glasshouse cultivations. The aim of this work was to study the effects of meteorological (temperature) and agronomical (herbicide) factors on the biology of the hemp aphid. In one experiment, hemp plants were kept at constant temperatures ranging from 20 to 30 °C, and aphid survival and fecundity were measured. In a related experiment conducted at 20 °C, plants were treated with field-appropriate rates of a selective graminicide containing quizalofop-P-tefuryl (40 gL^−1^, 4.38%, HRAC group 1), commonly used to control weeds in hemp, and aphid enzyme activity was measured in addition to population parameters. We found that hemp aphids could live, feed and reproduce within the whole studied range of temperatures, demonstrating its great evolutionary plasticity. However, the optimal temperature for development was 25 °C, at which the insect lived and reproduced for 25 and 15 days, respectively, with an average fecundity of 7.5 nymphs per reproduction day. The herbicide treatment increased the activity of superoxide dismutase (SOD), catalase (CAT), β-glucosidase, S-glutathione transferase (GST), oxidoreductive peroxidase (POD), and polyphenol oxidase (PPO) in the aphids, but only on certain days after treatment, which indicates a mild stress in aphid tissues, related to a higher reproduction and changed feeding behavior; aphids moved from the actively growing tips compared to untreated plants. The results of these experiments are discussed in terms of the impact on the future management of this pest.

## 1. Introduction

Aphids are pests of crops worldwide and are very sensitive to climate changes [1]. They have developed mechanisms of quick adaptation to changing environmental conditions. These are expressed in their morphology, biology and behavior [2,3,4]. Some researchers claim that aphids may serve as bioindicators of global warming [5]. An increase in temperature may be beneficial for them and promote their fecundity, but temperatures above 28 °C have been shown to cause negative effects such as dwarfing of individuals, reduced fecundity or life shortening [6]. This is because aphids (and other insects) are ectothermic animals, meaning they are strongly affected by thermal conditions [7,8,9,10,11,12]. The life table parameters used to analyze the dynamics of a population size and to estimate its growth and reproductive potential change with temperature [13]. Temperature limits or stimulates the activity of insects; its increase to the thermal optimum level accelerates their metabolism and results in longer feeding time, faster development, a higher number of generations and overall greater reproductive success [7]. Therefore, it is often possible to predict the outbreak of an arthropod pest, e.g., aphids in agricultural systems using temperature parameters [14]. Understanding the demography of an insect under different temperatures is the basis for its management in an eco-friendly manner [13]. 

Crop protection agents (insecticides, fungicides and herbicides), widely used in modern agriculture, often exert direct lethal effects on non-target organisms [15]. Insect populations are potentially exposed to large amounts of pesticides, but their doses greatly differ in space and time. Many insect individuals are being killed by insecticides, but others are subjected to a number of sublethal effects [16,17]. Although growers try to apply pesticides evenly at concentrations intended to kill target pests, many biotic and abiotic processes change the exposure to pesticides. In the field, the distribution of a pesticide within the plant canopy is always uneven and the compounds degrade over time [18]. Insects may also be directly or indirectly affected by other pesticides, including fungicides and herbicides [19]. Herbicides have been shown to inhibit or shorten the feeding period of insects, and thus reduce their population [20]. Herbicides may also be directly toxic to insects depending on the active ingredient formulation and dose of the chemical agent and the insect species [21,22]. Moreover, herbicides may induce plant defenses that increase resistance to insect herbivores. On the other hand, it has been shown that some herbicides positively affect insect pest populations, for example, the use of phosphonate herbicides decreased plant resistance and increased the number of aphids feeding on treated plants [23].

Numerous abiotic factors, such as temperature above the thermal optimum [24], treatment with pesticides or adverse response of the host plant [25,26] may lead to imbalance between the production of reactive oxygen species (ROS) and antioxidant processes in both plants and aphids [27,28]. The sudden increase in ROS levels is toxic to cells and leads to the destruction of proteins, lipids, and DNA [29,30]. However, aphids have evolved resistance mechanisms dealing with stressful conditions; they can neutralize the oxidation of biological molecules with antioxidant enzymes such as superoxide dismutase (SOD) and catalase (CAT) [31,32]. With detoxification enzymes such as β-glucosidase and S-glutathione transferase (GST), oxidoreductive peroxidase (POD), and polyphenol oxidase (PPO), they can also metabolize pesticides and plant phenols to less toxic compounds [25,33,34].

In the last decade, the intensive agricultural production of a small number of crops grown in monoculture has been highly criticized for its negative impacts on the environment. The global agricultural production has been narrowed to several crops. Taken together, the area of cereals exceeds 20% of global land area, or 61% of the total area of land cultivated [35]. In particular, rice, wheat, corn, barley, and millet are dominant over more than two thirds of the cropland of the world. Other leading food crops include sugarcane and oil palm, and cotton is the dominant crop grown for non-food usage. The recent policy of the European Union as well as the common worldwide trend greatly promote the broadening of cropping diversity and the restoration of numerous crops that have been abandoned. One such crop regaining the attention of the farmers is hemp (*Cannabis sativa* L.). It has been an important crop throughout human history, widely used for food, fiber, and medical purposes [36]. However, due to hallucinogenic properties of one of its metabolic compounds, Δ⁹-tetrahydrocannabinol (THC)—present in marijuana—the cultivation of this crop has been heavily restricted or banned in many countries. However, as hemp is such a multipurpose crop, there is potentially high value in its reintroduction to the arable cropping rotation worldwide. Recently, hemp has emerged as a source of highly valued, psychoactive, but non-hallucinogenic medicinal phytocannabinoids, such as cannabidiol (CBD), with distinct properties from marijuana [37]. Research studies have also shown other highly desirable properties of hemp, including antioxidant effects of its inflorescence extracts on oils containing unsaturated fatty acids, valuable for human health [38]. Apart from phytocannabinoids, hemp plants produce numerous terpenes that may also play important roles in human health, such as reducing inflammation and managing allergies and contribute to the entourage effect, that means they can modulate the psychoactive effect of the plant. Chemical methods, such as fast gas chromatography with flame ionization detection, permit simultaneous determination of terpenes and CBD in hemp [39], facilitating the studies of further improvements of these traits and maximization of the agronomic potential of this multifaceted crop [36].

Hemp crops are relatively free from pests and diseases, due to the content of terpenes which act as repellents to many insects, but also due to the limited cultivation. However, intensification of hemp production will most likely lead to the proliferation of species that interact with it. Hemp plants attract around 300 species of insects; to date only a few of them have been the cause of serious yield losses [40,41]. Pests that feed on aerial parts cause the greatest damage. In recent years, European corn borer, *Ostrinia nubilalis* Hbn., silver Y moth, *Autographa gamma* (L.) and European hop flea beetle, *Psylliodes attenuata* (Koch), have been responsible for significant damage to hemp grown in Europe [42]. Potential damage of flower buds and seeds is possible from Eurasian hemp borer, *Grapholita delineana* Walker. Of the sucking pests, white-flies, thrips and aphids can also cause serious damage to hemp plants [43]. In the USA, Cranshaw et al. [44] listed the hemp russet mite, *Aculops cannibicola* (Farkas), and aphids including *Phorodon cannabis* Passerini as the most threatening.

The aphids that are recorded as using hemp as host plants include *Phorodon cannabis* Passerini, *Aphis fabae* Scopoli, *Aphis gossypii* Glover, *Aulacorthum solani* (Kaltenbach), *Myzus persicae* (Sulzer) and *Rhopalosiphum rufiabdominale* (Sasaki) [45]. The species most responsible for significant damage is the hemp aphid *P. cannabis*, which has been found both in field and greenhouse cultivations [46]. It is a holocyclic and monoecious species, living on undersides of hemp leaves and on the flower stalks [47]. By piercing plant tissues and sucking plant liquids (mainly phloem), *P. cannabis* leads to plant wilting and yellowing, which seriously decreases crop yield [43]. This aphid is also reported to transmit Cannabis Streak Virus [48]. The distribution of *P. cannabis* extends from central, eastern and southern Europe into Turkey and great parts of Asia and North Africa. In 2016, the hemp aphid was introduced to North America. It was first found in Colorado and it is now widespread throughout that state [46], Oregon (https://www.oregon.gov/oda/shared/Documents/Publications/IPPM/CannabisAphidAlert.pdf accessed on 15 March 2021) and several other US states, including California (https://blogs.cdfa.ca.gov/Section3162/wp-content/uploads/2019/09/Phorodon-cannabis.pdf accessed on 15 March 2021) as well as some parts of Canada [46].

The knowledge of hemp aphid biology is still incomplete and insufficient to facilitate optimized crop protection strategies. The aim of this study was to find out how the *P. cannabis* responds to selected abiotic factors. In particular, we were interested in abiotic factors that cause suboptimal conditions for aphids such as the rise in temperature by a few up to 10 °C and the indirect effects of pesticides.

In this study, we assessed the effects of temperature rise likely via climate warming on the survival, development, fecundity and the life demographic parameters of the *P. cannabis*. We also assessed the effect of a commonly used graminicide on *P. cannabis* fecundity. We measured whether these mild abiotic changes affect the activity of defense response enzymes in aphids, bearing in mind that fast and coordinated interaction of antioxidative (SOD, CAT), oxidoreductive (PPO, POD), and detoxification (GST, β-glucosidase) enzymes enables the neutralization of ROS and harmful metabolites. We hypothesized that the change in sap composition affects enzymatic markers in aphid tissues. The strength and duration of this reaction and its developmental consequences were unpredictable and required experimental studies, which we performed using the methods listed below.

## 2. Materials and Methods

### 2.1. Insects

Hemp aphids (*P. cannabis*) were collected from a field of hemp (*C. sativa*) cultivar Henola (Institute of Natural Fibers and Medicinal Plants, IWNiRZ, Poznan, Poland). The field was located in Cerekwica (52°31′28.9′′ N 16°41′16.6′′ E) in the region of Great Poland. Infested plants were transferred to a growth chamber (Mytron Bio- und Solartechnik, Heiligenstadt, Germany) and kept under controlled conditions (20 ± 1 °C, 65 ± 5% humidity, and 16L:8D photoperiod). The aphids were supplied with new, healthy hemp plants of cv. Henola. For the experiments, apterous parthenogenetic females were used.

### 2.2. Host Plants

Hemp seeds of cv Henola, (IWNiRZ, Poznan, Poland) were sown into pots with potting soil TS1-876, pH 6.0 (Klasmann-Deilmann Polska Sp. z o.o, Warsaw, Poland). Plants were grown in a growth chamber (Mytron Bio- und Solartechnik, Heiligenstadt, Germany) for six weeks and watered regularly. The photoperiod was 16L:8D with day and night temperatures of 23 ± 1 °C and 19 ± 1 °C, respectively, and a relative humidity of approximately 65 ± 5%. Experiments were done using 6 week-old plants.

### 2.3. Effect of Temperature on Survival, Development, Fecundity and Demographic Parameters of Phorodon cannabis

The experiment was carried out in growth chambers (Mytron Bio- und Solartechnik, Heiligenstadt, Germany)) at the following temperatures: 20, 25, 28, and 30 °C, with standardized humidity (65 ± 5%) and photoperiod (16L:8D). For each temperature, 40 apterous adult females of *P. cannabis* applied per plant. The next day, the females were removed, leaving 100 nymphs, which were subjected to further analysis. The nymphs were counted each day until they were mature. For each temperature treatment, the percentage mortality of nymphs was determined.

In a related experiment in the same conditions, 50 apterous females, 1 day after reaching maturity, were placed separately on 6-week-old hemp plants (10 aphids per plant). The length of the following developmental stages was recorded: pre-reproduction, reproduction, post-reproduction and total life span. In order to calculate the fertility of females, newly born nymphs were counted and removed once per day with a soft brush. The experiment continued until all aphids died. Demographic parameters for *P. cannabis* populations at the four different temperatures were determined according to the methods described by Birch [49]. The net reproduction rates (Ro), the mean generation time (T), the intrinsic rate of increase (rm), the finite rate of increase (λ) and the time necessary for the population to double (DT) were calculated. The intrinsic rate (rm) was calculated using the Wyatt and White formula [50]: rm = 0.738 (lnMd)/d, where d is the period from birth to the first nymph produced (reproduction) and Md is the number of nymphs born in the period from time d. The net reproduction rate is given by Ro = ∑ (mxlx), where lx and mx represent cumulative daily survival and fertility, respectively; mean generation time T = lnRo/rm; the finite rate of increase l = erm and doubling time DT = ln2/rm.

### 2.4. Effect of Herbicide on the Aphid Population

The effect of herbicide was studied using the systemic graminicide Pantera 040 EC (Arysta LifeScience, Amsterdam, the Netherlands) containing the active substance quizalofop-P-tefuryl at the concentration 40 gL^−1^ (4.38%). The compound is the derivative of arylphenoxypropionic acids, FOPs) According to the Herbicide Resistance Action Committee (HRAC), the product belongs to the group A, which is responsible for the inhibition of Acetyl CoA carboxylase (https://hracglobal.com/tools/classification-lookup accessed on 23 March 2021). The herbicide is used for controlling monocotyledonous weeds in dicotyledonous crops. In Poland, the product is officially registered graminicide. Pantera 040 EC is a systemic herbicide, which is very quickly absorbed by the leaves of annual and perennial grass weeds. The active substance translocates throughout the plant and the product accumulates at the growth tissues of shoots and rhizomes, where it blocks the synthesis of lipids, leading to plant death. The first symptoms are visible after 6–10 days and a full effect is observed 14–20 days after treatment. The period between the last application of the product to the next sowing or planting of succeeding crops is 120 days.

The compound was diluted in distilled water to the final concentration of 0.04%, exactly as it is recommended by the producer (Arysta LifeScience, Amsterdam, the Netherlands) to the farmers using Pantera 040 EC in the field. The solution was applied on stems and leaves of 6-week-old hemp plants with a soft brush. There were four variants of the herbicide application: (1) the whole plant, (2) the upper part of the plant (1/3 from the top), (3) the middle part of the plant (1/3), and (4) the bottom part of the plant (1/3 of the plant from the bottom). Distilled water was applied in the same way to the control plants. There were 9 replicates (plants) per each treatment. One day after herbicide application, 100 wingless females of *P. cannabis* were delicately placed on the herbicide-treated plants using a soft brush. Plants with aphids were transferred to the growth chamber and kept in the previously described conditions, i.e., 16L:8D photoperiod, 23 ± 1 °C (day), 19 ± 1 °C (night), 65 ± 5% humidity). Observations were made 3, 5 as well as 7 days after the application of aphids. Females and nymphs were counted on all plants. The preferred habitat (plant fragment) of aphids and their movement were observed.

### 2.5. Effect of Herbicide on the Enzymatic Activity in Aphid Bodies

Studies of the aphid enzymes followed the methods described by Dampc et al. [24]. The experiments were carried out in a growth chamber with photoperiod, temperature and humidity regimes described in Section 2.1.

Hemp plants were treated with herbicide (Pantera 040 EC at a concentration of 0.04%) or water as described in 2.4. The next day, 35 aphids were applied to each plant treatment using a soft brush. Aphids were left to feed for 24, 48, 72, 96, 168 (7 days) and 240 h (10 days). The experiment was replicated three times; each replicate was composed of 3 plants per variant (treatment × feeding time). Immediately after removal from the plant, the aphids were frozen with liquid nitrogen and then stored in a deep freezer at −80 degrees (ULT 2186 U-O-C, Rheem Manufacturing Company, Scientific Production Division, Asheville, NC, USA).

Aphids (35 individuals) from each treatment were placed in a tube containing phosphate buffer (0.1 M, pH 7.0) and were homogenized at 0 °C and centrifuged at 4 °C using an Eppendorf Centrifuge 5810 R (Eppendorf, Hamburg, Germany). The supernatant was used for the subsequent determination of enzymatic activity. All biochemical assays were performed in three independent biological replicates (*n* = 3) for each enzyme, and for each time period (0, 24, 48, 72, 96, 168, and 240 h).

The content of proteins in homogenates prepared from aphid tissues was measured according to Lowry [51]. The preparation of the calibration curve was done using the solution of the core albumin (Sigma-Aldrich, Saint-Louis, MO, USA). The content of proteins was determined in mg·mL^−1^.

The activity of superoxide dismutase (SOD) was measured according to Wang et al. [52]. In order to determine the activity of catalase (CAT), we used the modified method of Aebi [53]. The β-glucosidase activity was determined by the hydrolysis reaction of *p*-nitrophenyl-β-D-glucopyranoside with β-glucosidase, as described by Katagiri [54]. The measurement of glutathione S-transferase (GST) activity was done according to Leszczyński [55]. The activity of polyphenol oxidase (PPO) was measured according to Miles [56] with a modification published by Laurema [57], while the activity of peroxidase POD followed the method described by Fehrman [58].

### 2.6. Statistical Analyses

The statistical analyses were done with data analysis software systems: Statistica ver. 13 (TIBCO Software USA Inc., Palo Alto, CA, USA; 20 March 2017, http://statistica.io). All data were tested for normality using the Shapiro–Wilk test, and Lilliefors and homogeneity of variances using the Levene test. Tabulated results are attached in the Supplementary Materials (Table S1).

The duration of the pre-reproductive, reproductive and post-reproductive periods, life span, fecundity at the different temperatures and the population growth were analyzed using one-way analysis of variance (ANOVA) applying a significance level α ≤ 0.05 followed by post hoc Tukey’s tests to determine differences among the treatments. The differences between the average enzymatic activities in the tissues of *P. cannabis* in the treatments were analyzed using two-way analysis of variance (ANOVA). Herbicide and feeding time (0, 24, 48, 72, 96, 168, and 240 h) were fixed factors. Post hoc comparisons were undertaken using Tukey’s HSD test.

## 3. Results

### 3.1. Effects of Temperature on Aphid Development, Fecundity and Mortality

This research indicates that temperature had a significant effect on the development of hemp aphid. As temperature increased, the duration of the pre-reproductive period decreased, ranging from an average of 11 days at 20 °C to 5.4 days at 30 °C (Table 1). Statistically significant differences were found between the development of nymphs at all tested temperatures, in the range between 20 and 30 °C. The reproductive period was also significantly longer at lower temperatures. The average duration of this period was c. 15 days at 20 and 25 °C, constituting 60% of the total lifespan of the aphids. However, at 28 and 30 °C, this was significantly reduced; the reproductive period at 30 °C lasted only two days, just 14% of the hemp aphid lifespan (Table 1). The average post-reproductive period ranged from 3 days at 25 °C to 7 days at 30 °C. On the other hand, at 20 °C, females died immediately after reproduction (Table 1). The total aphid life span ranged on average from 15 to 26 days and decreased with increasing temperature. The lifespan of aphids maintained at 20 and 25 °C differed significantly from that of females kept at 28 and 30 °C (Table 1).

Temperature significantly influenced all phases of the hemp aphid life (prereproduction (F_(3,172)_ = 2178.39, *p* < 0.01; reproduction (F_(3,172)_ = 55.22, *p* < 0.01; postreproduction (F_(3,172)_ = 41.49, *p* < 0.01). It was also an important factor in changing the overall lifespan and fecundity of aphids (lifespan (F_(3,172)_ = 30.95, *p* < 0.01; fecundity (F_(3,172)_ = 144.64, *p* < 0.01). Significant statistical differences were demonstrated between the fertility of aphids living at all temperatures. The highest fertility (on average 112 nymphs per female) was found at 25 °C. However, after exceeding this level of fertility, a clear reduction was observed, down to the mean of 2 nymphs per female at 30 °C (Table 1).

**Figure 1:**
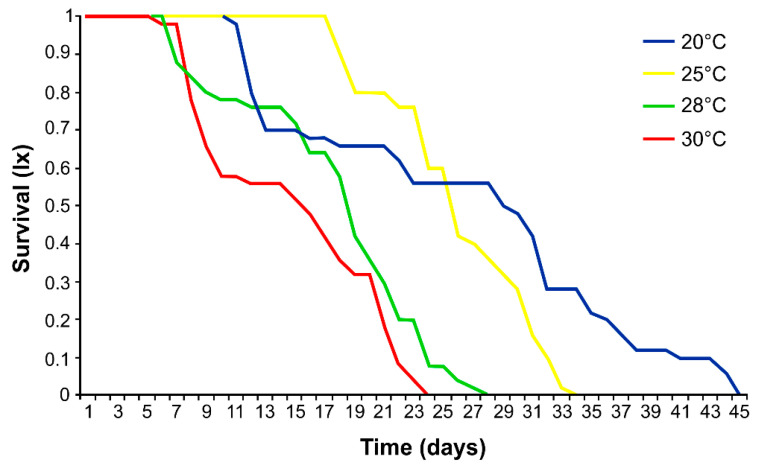
Effect on survival rates of apterous *Phorodon cannabis* (*n* = 50 individuals at each temperature: 20, 25, 28, 30 °C).

**Figure 2:**
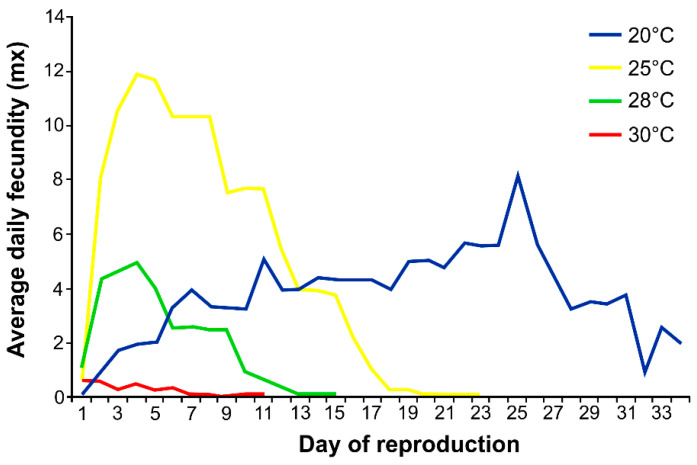
Number of nymphs produced (fecundity) daily by apterous *Phorodon cannabis* at different temperatures (*n* = 50).

At 20 °C, the nymph’s survival was high (98%). At 25 °C, nymph survival decreased to 71%, at 28 °C it decreased to 61%, and at 30 °C only 43% of nymphs reached maturity. Temperature also affected the survival of mature aphids. The increase in temperature from 20 to 30 °C reduced aphid survival by almost half (Figure 1). Temperature also affected the daily fecundity of female aphids (Figure 2). The highest mean daily fecundity was achieved on Day 4 of the reproduction for females kept at 25 °C (approximately 12 nymphs per female).

The intrinsic rate (rm) of population increase was highest (0.355) at 25 °C and lowest (0.019) at 30 °C (Table 2). During one day of feeding on *C. sativa* host plants, the hemp aphid population increased (λ = finite rate of increase) on average from 1.017 (30 °C) to 1.426, peaking at 25 °C. The average development time of one generation (T) ranged from 8 days at 30 °C to 21 days at 20 °C. The population increased (Ro) from 1.2 to 58 times, and the peak was also found at 25 °C. These results demonstrate that the population of hemp aphid *P. cannabis* achieves the best demographic parameters at 25 °C.

### 3.2. Effect of Herbicide on the Aphid Population

**Figure 3:**
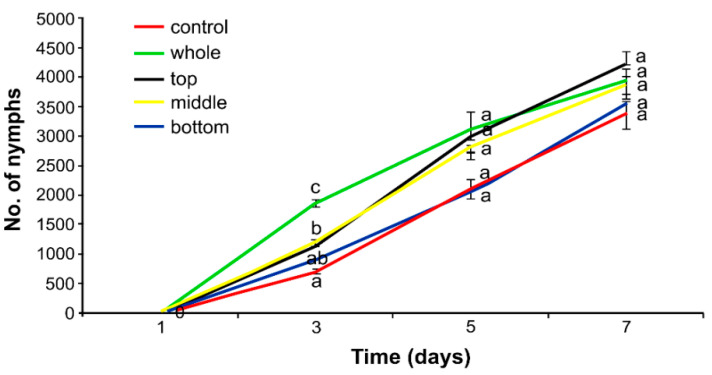
Cumulative increase in the number of hemp aphid (*Phorodon cannabis*) on hemp (*Cannabis sativa*) plants after application of Pantera 040 EC graminicide to different sections of the plant (mean ± SE; control = water). Values not followed by the same letter are significantly different at the level of *p* < 0.05 (Tukey multiple range test).

Differences were found in the number of nymphs produced by aphids feeding on *C. sativa* treated with the herbicide compared to the control (aphids feeding on plants without herbicide application). The tendency of relationships was the same over the whole experiment period; however, on Day 3, statistically significant differences were observed (Figure 3). On plants treated with the herbicide along the whole stem and on all leaves at the same time (bottom, middle and the top of the plant), on Day 3, female hemp aphids produced 2.6 times more nymphs as compared to the controls, untreated with the herbicide. When the middle and top parts of hemp plants were treated with the herbicide, there were 1.7 and 1.6 times more nymphs than in the controls, respectively. On plants treated only at the bottom part, the population growth was comparable to that of the controls (Figure 3).

**Figure 4:**
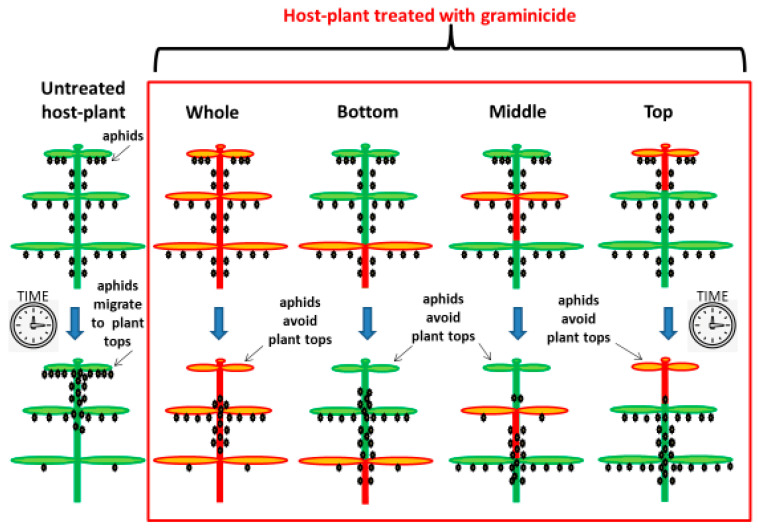
Scheme of the migration patterns of nymphs and adult hemp aphids (*Phorodon cannabis*) after herbicide application to the whole plant or individual parts of the host plant (top, middle or bottom third of the plant) (red box) and control plants untreated with the herbicide (left part of the scheme). The upper row shows the position of aphids at the beginning of the experiment (even distribution along the plant), and the bottom row shows the position of aphids 7 days later, i.e., 7 days after plant treatment (top of the plant in untreated variant; bottom and middle in the herbicide treated variant). The herbicide used in this study was Pantera 040 EC, the selective graminicide containing quizalofop-P-tefuryl (40 g·L^−1^, 4.38%, HRAC group 1: inhibition of Acetyl CoA carboxylase), commonly used to control weeds in hemp (*Cannabis sativa* L.) in Poland. Aphids were applied to the host plants one day after treatment.

In all experimental treatments, the migration of adult aphids and their nymphs along the plants was observed. On plants treated with herbicide, the aphids most often colonized the stems, and no insects were observed on the tops of shoots. In contrast, on control plants, *P. cannabis* was observed feeding both on leaves and on stems, seemingly preferring to settle within the top section of the plants. Seven days after the start of this experiment, the drying up and dying of the plant tops was observed on control plants only, due to intensive colonization and feeding by aphids. This was not the case on plants treated with the herbicide, where feeding on plant tops was avoided (Figure 4).

### 3.3. Effect of Herbicide on the Aphid Enzyme Activity

#### 3.3.1. Superoxide Dismutase (SOD) and Catalase (CAT) Activity

**Figure 5:**
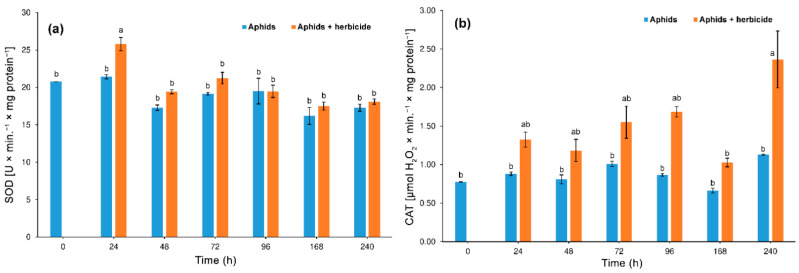
Activity change of superoxide dismutase (SOD) (**a**) and catalase (CAT) (**b**) in the tissues of hemp aphid (*Phorodon cannabis*) hemp plants (*Cannabis sativa*) following the application of Pantera 040 EC graminicide (mean ± SE; control = aphids on untreated *C. sativa*). Values not followed by the same letter are significantly different at the level of *p* < 0.05 (Tukey multiple range test).

We found that herbicide had no significant effect on SOD activity in aphid tissues (two-way ANOVA F_(1,28)_ = 3.92, *p* > 0.05). However, the influence of time was significant (two-way ANOVA F_(6,28)_ = 4.89, *p* < 0.001). There was no significant interaction between herbicide and time in SOD activity (two-way ANOVA F_(6,28)_ = 1.56, *p* > 0.05). The colonization of hemp plants treated with the herbicide caused an increase in superoxide dismutase activity in aphid tissues (Figure 5a). The highest and statistically significant level of SOD activity was demonstrated after 24 h of feeding activity on plants treated with the herbicide compared to aphids feeding on untreated plants. At all time intervals (except 96 h), higher SOD activity was observed in the tissues of aphids feeding on the herbicide-treated plants. It was also found that SOD activity in *P. cannabis* tissues alternately increased and decreased during the course of the experiment. Two days (48 h) after the start of aphid feeding on herbicide-treated plants, the activity of SOD in insects was similar to the activity of this enzyme in insects feeding on untreated plants.

The results of our research showed a significant influence of herbicide on CAT activity in aphid tissues (two-way ANOVA F_(1,28)_ = 15.95, *p* < 0.001). Moreover time had a significant effect on the activity of CAT (two-way ANOVA F_(6,28)_ = 3.37, *p* < 0.001). There was no significant interaction between herbicide and time in CAT activity (two-way ANOVA F_(6,28)_ = 1.2, *p* > 0.05). *P. cannabis* feeding on plants treated with the herbicide induced hydrogen peroxide-decomposing enzyme (CAT) activity. The levels of CAT activity increased up to 96 h, then decreased on Day 7, but on Day 10 the most significant increase in CAT activity was observed (Figure 5b). There was no significant increase in CAT activity in aphids feeding on control plants.

#### 3.3.2. β-Glucosidase and S-Glutathione Transferase (GST)

**Figure 6:**
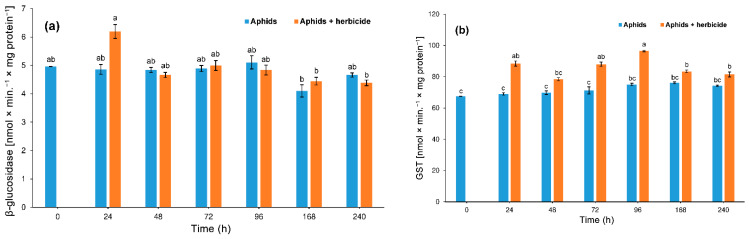
Activity change of β-glucosidase (**a**) and s-glutathione transferase (GST) (**b**) in the tissues of *Phorodon cannabis* after the application of Pantera 040 EC graminicide to the hemp plant (mean ± SE; control = aphids on the *Cannabis sativa*). Values not followed by the same letter are significantly different at the level of *p* < 0.05 (Tukey multiple range test).

In our study, the application of the herbicide had no significant effect on β-glucosidase activity in the aphid (two-way ANOVA F_(1,28)_ = 0.91, *p* > 0.05). However, the influence of time was significant (two-way ANOVA F_(6,28)_ = 3.53, *p* < 0.01). There was no significant interaction between the herbicide and time (two-way ANOVA F_(6,28)_ = 1.08, *p* > 0.05). Only after 24 h of aphids feeding on herbicide-treated plants did the application of the herbicide cause a significant difference (increase) in β-glucosidase activity in hemp aphid *P. cannabis* tissues compared to the controls. After this time, a gradual decrease in the activity of this enzyme in the tissues of insects was observed (Figure 6a).

The activity of GST in the tissues of the aphids feeding on the plants treated with the herbicide differed significantly from the activity of this enzyme in the aphids feeding on the control plants without the application of the herbicide. The GST activity found in aphid tissues differed significantly depending on herbicide (two-way ANOVA F_(1,28)_ = 92.4, *p* < 0.001) and time (two-way ANOVA F_(6,28)_ = 12.71, *p* < 0.01), and there was significant interaction between herbicide and time in GST activity (two-way ANOVA F_(6,28)_ = 6.08, *p* < 0.01). A gradual increase in GST activity was observed in the tissues of aphids inhabiting plants treated with the herbicide up to 96 h of feeding, and then it decreased. The highest level of GST was found in tissues of aphids feeding on hemp plants treated with the herbicide for 24 and 96 h (Figure 6b).

#### 3.3.3. Polyphenol Oxidase (PPO) and Peroxidase (POD)

**Figure 7:**
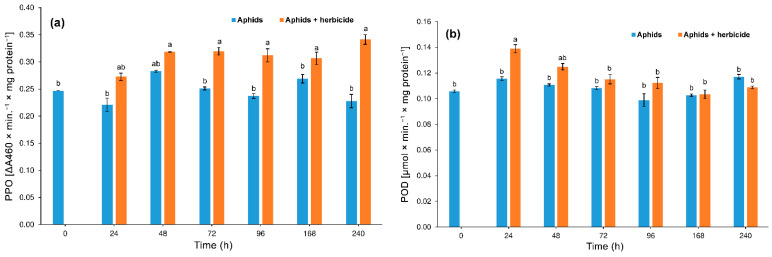
Activity change of polyphenol oxidase (PPO) (**a**) and peroxidase (POD) (**b**) in the tissues of *Phorodon cannabis* after the application of Pantera 040 EC graminicide to the hemp plant (mean ± SE; control = aphids on the *Cannabis sativa*). Values not followed by the same letter are significantly different at the level of *p* < 0.05 (Tukey multiple range test).

Both the herbicide (two-way ANOVA F_(1,28)_ = 42.86, *p* < 0.001) and time (two-way ANOVA F_(6,28)_ = 3.56, *p* < 0.001) significantly affected the activity of PPO. Our research has shown a significant interaction between herbicide and time in PPO activity (two-way ANOVA F_(6,28)_ = 2.64, *p* < 0.001). The activity of polyphenol oxidase significantly increased in tissues of aphids feeding on plants treated with the herbicide after 72 h of aphid feeding. The highest activity of PPO in the tissues of the aphids inhabiting plants treated with the herbicide as compared to the aphids feeding on herbicide-untreated plants was recorded on Day 10 of feeding (Figure 7a).

Herbicide treatment significantly affected POD activity present in aphid tissues (two-way ANOVA F_(1,28)_ = 4.49, *p* < 0.001). Also, time had a significant effect on the activity of POD (two-way ANOVA F_(6,28)_ = 6.22, *p* < 0.001). Moreover, there was a significant interaction between herbicide and time of POD activity (two-way ANOVA F_(6,28)_ = 2.74, *p* < 0.001). The POD activity in the tissues of aphids feeding on plants treated with the herbicide increased significantly soon after the start of their feeding (24 h). Thereafter, a gradual decrease in this enzyme activity was detected until 168 h and then a slight increase after 10 days was noted. The POD activity in the tissues of the aphids feeding on herbicide-treated plants was significantly different from that in the tissues of the aphids feeding on control plants only at 24 h of feeding (Figure 7b).

## 4. Discussion

### 4.1. Effect of Temperature on Survival, Development, Fecundity and Demographic Parameters of Phorodon cannabis

This study demonstrates a high evolutionary plasticity of hemp aphid *P. cannabis*. The insect could survive, thrive, and reproduce at temperatures ranging from 20 to 30 °C. The life span was longest between 20 and 25 °C (*ca*. 25 days on average), and at this temperature range the reproductive period was also longest (*ca*. 15 days on average). However, at 20 °C the pre-reproduction period was longer than at 25 °C (11 days and 6.4 days, respectively), and at 20 °C the aphids died immediately after reproduction ceased, whereas at 25 °C the mean post-reproduction period was 3 days. At both temperatures, the fecundity of hemp aphids was abundant, varying from ca. 55 nymphs at 20 °C to ca. 112 nymphs at 25 °C with a daily fecundity rate of 3.7 and 7.5 nymphs/day, respectively. Considering the similar reproduction period at these two temperatures (15 days) an increase in temperature by 5 °C increased aphid fecundity twice. However, further temperature increase caused a decrease in hemp aphid longevity by 6.4 d at 28 °C and by 3.1 d at 30 °C. A similar effect at 28 °C was observed in the apple aphid, *Aphis pomi* (De Geer), with an associated peak in the activity of enzymes indicating stress in aphids [24]. In our study, the temperature increase above 25 °C significantly shortened the pre-reproduction period, by 0.4 and 0.6 day at 28 and 30 °C, respectively, and the reproduction period also decreased significantly, by 5.6 and 7.5 days. The same phenomenon—a significant reduction—concerned the longevity of aphid life, by 6.4 and 3.1, respectively, at 28 and 30 °C. The post-reproductive period at 28 °C was comparable to that at 25 °C, but a further increase of only 2 °C dramatically decreased this time to 7.6 days on average, so it was nearly three times longer at 25 °C than at 28 °C, thus reducing the species permanence. Moreover, the daily fecundity dropped down significantly at 28 °C to less than three nymphs per day. At 30 °C, the reproductive output was only 1 nymph per day of the reproduction period and the procreation time was only 2 days in the lifetime of hemp aphid. This substantial decrease was also highly significant. Summarizing, the temperatures between 20 and 25 °C were optimal for the development of hemp aphid *P. cannabis*, whereas 28 °C and above still enabled aphids to live and procreate, but the demographic parameters were much less as compared to the lower temperatures used in this study.

Hemp aphid (*P. cannabis*) was first described in Poland in 1914 [59]. Most probably it accompanied the production of hemp, which was commonly grown at that time, as one of the best domestic fiber crops (as well as linseed). The abrupt decline of hemp cultivation caused by drug abuse and the subsequent legal restrictions regarding its cultivation led to the disappearance of this crop; for over half a decade there were only several fields in Poland. The insect is monophagous and highly specialized to hemp but has persisted, probably also by feeding on wild and cultivated hop (*Humulus lupulus* L.) [47]. Immediately after the re-appearance of hemp fields in the agricultural landscape, the *P. cannabis* population has been restored, although the acreage of hemp is still very small.

The hemp aphid is usually regarded as typical of warm zones [46]. However, cold climates, such as those experienced in Poland in the period 1779–2000, when the average yearly temperature was 7.7 °C, apparently provided sufficient thermal conditions for this insect. One may speculate that the hemp aphid population living in the cold weather typical of Poland until the last two decades is probably more adapted to cold weather compared to hemp aphid populations feeding on hemp plants growing in much hotter climates, such as Afghanistan or Uganda. Further studies are needed to validate this hypothesis. The last 40 years have been the warmest period in the 230-year history of Poland since records began, with average annual temperature increases of +8.7 °C, +8.9 °C, +9.2 °C and +9.3 °C recoded for the past 4 decades (http://klimada.mos.gov.pl/ accessed on 23 March 2021). In 2017 and 2018, at three sites in Poland, the mean temperatures in the summer months (June to September) ranged from 18.1 to 22.2 °C [60]. In Poland, at these temperatures, hemp plants in the field are fast growing, soft and full of phloem sap. The usual temperature in glasshouse cultivation is close to 25 °C. This temperature coincides with the optimal thermal conditions for the development and reproduction of hemp aphids in our study.

The geographic distribution of insects highly depends on their plasticity in response to temperature conditions. In most cases, this thermal range is relatively low and both increased and decreased temperature shifts strongly affect insect survival and activity [61]. Insects found in temperate places can thrive within a broader temperature range than their tropical counterparts [62]. Conversely, species living in the tropics may by more sensitive to temperature changes as compared to those living in cooler zones [63]. In our study, we found that the life parameters of hemp aphids at 20 °C differed compared to warmer temperatures; the longer the aphid life, the higher its fecundity. However, the lifespan and aphid fecundity at 30 °C were highly restricted In Poland the number of hot days is not high, so‒by now‒hemp aphids could live and develop in optimal conditions.

Most of the species on Earth cannot regulate their own body temperatures metabolically, which is one of numerous causes of mass human-induced species extinction via climate change [64]. Kellermann et al. [62] showed that specialist insect species may lack genetic variation in key traits, making them vulnerable to conditions beyond the temperature range they experienced throughout numerous generations. In contrast, generalist insect species are less likely constrained in their responses to thermal variation and this plasticity enables them to better adapt to changes in climate. Aphids are known to be highly plastic, able to adapt to different environmental conditions [2]. Although the hemp aphid is a specialist, based on the results of our study we can speculate that hemp aphids living in temperate regions will benefit from moderate global warming and could expand to new territories in the north, similar to other aphids [65]. Due to its high plasticity, the success of this monophagous species mainly depends on the cultivation of its host plant and continued restriction for hemp cultivation may be the only cause of keeping the insect constrained to current habitats.

A small increase in temperature favors the faster development of insects [7]. On the other hand, the shorter nymphal stages observed during our research on *P. cannabis* result in a shorter time frame for the predators and parasitoids to attack the aphid pest. This can contribute to increasing its survival rate and thus its reproductive success. Parasitism could be reduced if host populations emerge and pass through vulnerable life stages before parasitoids emerge [66]. However, it has been shown that aphids at elevated temperatures have a weaker response to the alarm pheromone released when threatened by a predator, which may contribute to greater mortality via predators [7].

Insects are affected by the microclimatic conditions created by the surrounding landscape. Homogenous landscapes are warmer than more complex landscapes [67]. Changes in land use, mainly caused by human activity and intensive agricultural production, can seriously affect the thermal tolerance and adaptability of these insects. According to Alford et al. [68], aphids from simple landscapes could possess an enhanced thermal tolerance in response to greater temperature variation. In contrast, aphids inhabiting plants in complex landscapes may be more tolerant to cold. The authors suggest that in conjunction with global warming, aphids will be less exposed to cold weather stress, especially in the spring months [68]. They also believe that global warming may increase the adaptation of insects from the temperate zone, which also includes the hemp aphid. This species has been recorded in Poland for more than 100 years, which proves its adaptation to a temperate climate. The results of our research indicate the plasticity of hemp aphid and the possibility of its development even in unfavorable temperatures.

### 4.2. Hormetic Effect of the Herbicide on Hemp Aphid Population

According to Kraus and Stout [19], there are several ways that herbicides can affect insect herbivores, comprising direct and indirect effects. The immediate effect of the herbicide application may cause direct mortality of insects present in the field during the chemical spray or feeding on plants soon after. However, herbicides may also trigger plant defense responses that increase resistance to insect herbivores, or they may alter the quantity and composition of weed populations [19]. Although this topic is fascinating and has a high connection to common agricultural practice, not many experiments have previously investigated the effect exerted by herbicides on aphids. The study on the influence of glyphosate—a non-selective herbicide—on the rose-grain aphid *Metopolophium dirhodum* (Walker) clearly showed a negative effect on aphid population parameters [69]. The effect was progressive with the increasing concentration of the herbicide, and the difference between the untreated control and the high dose of the herbicide (133.8 mmol dm^−3^ of the active ingredient) was of two orders of magnitude. The population of the Russian wheat aphid (*Diuraphis noxia* Kurdjumov)—one of the most important pests of wheat worldwide—was similarly significantly reduced by fenoxaprop-p-ethyl with fenohlorazole-ethyl, tribenuron-methyl and 2,4-D ester, which are the active ingredients in some herbicides [67]. This effect was continuously observed from the third day of herbicide application until the end of the experiment (Day 21). Similarly, chlorophenoxyacetic herbicides (4-chloro-2-methylphenoxyacetic acid, MCPA and 2,4-dichlorophen oxyacetic acid, 2,4-D) used for weed control in cereals affected the probing behavior of the grain aphid, *Sitobion avenae* F., bird cherry-oat aphid, *Rhopalosiphum padi* (L.), and the rose-grain aphid, *M. dirhodum*, by inhibiting the uptake of phloem sap [20]. These studies indicated that some herbicides can not only clear the field of weeds but also reduce the populations of insect herbivores, including aphids.

Some studies on the effects of abiotic factors on aphids concerned other xenobiotics. Łukaszewicz and collaborators [70] studied the effect of the selenium enrichment of pea plants on the pea aphid, *Acyrthosiphon pisum* (Harris), and found that the application of 10 and 20 µM sodium selenite changed aphid feeding behavior; the plant treatment reduced the activity of aphids associated with the phloem phase and extended the non-probing time. Due to the decrease in food availability, reproduction was reduced and significantly limited demographic parameters of the pea aphid population. The activity of antioxidant, detoxifying and redox enzymes in the tissues of *A. pisum* increased, but the damage to the aphid bodies was irreversible [70]. The bio-fortification of pea plants with selenium, therefore, could give a double benefit to farmers. When *A. pisum* was feeding on lead-treated pea plants, the number of pea aphids reaching the phloem sap was greatly suppressed [71]. The results of our study are totally opposed to the examples presented above, but our experiment was done using a graminicide, i.e., it is relevant to the real-world scenario regarding gramineous weed control in the dicot hemp crop. We found that hemp aphids, *P. cannabis*, did not attempt to move off treated host plants and, moreover, hemp aphid fecundity doubled. We attribute this effect to hormesis.

Over many years, the toxicological model was assuming that dose–response is a continuum [72]. In contrast, Calabrese [73] introduced the concept of hormesis, in which high- and low-dose effects considerably differ, with the former causing stress and inhibition and the latter being a stimulant of many processes [74,75]. As such, a biological phenomenon of hormesis emerged as a fundamental dose–response model of numerous factors, not only chemical (toxicology) but also physical, and biological. Moreover, many researchers advised to include a time component [76,77], ideally multiple time-points, to obtain a full picture of the studied effect, as in our experiment. This revealed that the hemp aphid population considerably increased after graminicide application compared to untreated controls. This general trend was observed over the whole study (7 days), but statistically significant differences were found on the third day of feeding on graminicide-treated plants. The most reasonable explanation of this phenomenon is the presence of quizalofop-P-tefuryl in phloem sap, soon after plant treatment. Presumably, this active ingredient of the graminicide or other substances added to the Pantera 040 EC preparation stimulated hemp aphids to give birth to more nymphs. Then, with time, the concentration of the xenobiotics decreased. In future experiments, we plan to check this hypothesis.

A small amount of the xenobiotic, previously never encountered in phloem sap, apparently caused a mild stress in aphids, indicated by the activity of superoxide dismutase, which increased considerably in aphid bodies 24 h after feeding on herbicide-treated plants, and then decreased (but its activity was higher than in controls at all following time-points of the study). The activity of catalase also increased significantly 24 h after aphid feeding commenced on phloem sap containing traces of the graminicide, then the activity of this enzyme was maintained at a high level and peaked on Day 10. This reaction was relatively long after herbicide application. Similar results were obtained by Urbańska [78], who also observed that the activity of SOD came first in the response of bird cherry-oat aphid, *R. padi*, to stress caused by a changed diet. Soon, the activity of this enzyme decreased, whereas the activity of catalase remained high long after stress, showing the specific response of the aphid to the increasing concentrations of hydrogen peroxide [78]. An increased and sustained CAT suggests that aphids actively respond to stress by reducing hydrogen peroxide with the catalase. Both SOD and CAT serve as antioxidant enzymes neutralizing the oxidation of biological molecules. In the mosquito *Anopheles gambiae* Giles, the detoxification of reactive oxygen species by catalase resulted in increased insect fecundity [79]. A systemic reduction in catalase activity by ds RNA-mediated knockdown significantly decreased the reproduction of mosquito females, indicating that catalase plays a central role in protecting the oocyte and early embryo from ROS damage. In our study, high activity of catalase coincided with a raised fecundity of hemp aphids, which suggests that the mechanism described by DeJong and collaborators [79] may apply to many groups of insects. 

In our study, the detoxification enzymes such as β-glucosidase and S-glutathione transferase (GST) showed increased activity in the bodies of hemp aphids feeding on the phloem sap of plants treated with herbicide. Again, the activity of one of the enzymes (β-glucosidase) increased significantly only 24 h after the beginning of feeding on graminicide-treated plants, whereas the significant increase in GST activity lasted over the whole period of the experiment. This reaction differed from the enzymatic defense response of apple aphid, *A. pomi*, but the measurements in the study on apple aphid concerned the temperature increase, and not the herbicide [24]. However, there was a similarity in β-glucosidase activity fluctuations in the response of pea aphid, *A. pisum*, feeding on a low dose of selenate-treated pea plants, and GST activity changes to a high dose of selenate [70].

In this experiment, the oxidoreductive enzymes POD and PPO, able to metabolize pesticides as well as plant phenols to less toxic compounds, also differed from each other with respect to their activity in aphid bodies, with PPO being active only in the first 24 h and POD being active during the whole experiment. Dampc et al. [24] showed a similar response of POX and PPO activity in the pea aphid to host plant treatment with selenate, but the enzymatic reaction was delayed compared to our study, with the peak activity 48 and not 24 h after treatment. Presumably, the delayed effect to selenate treatment was caused by a longer uptake of nutrients from the soil, as compared to the direct application of the graminicide to stems and leaves of the above-ground part of the host plant, as in our experiment. Again, the enzymatic response of *A. pomi* took a different course, with a small increase up to Day 4 and sudden jump between Day 4 and Day 14, but that study concerned the effect of temperature increase on PPO and POD enzyme activity in aphids [24]. Apparently, the reactions to thermal stresses and to the changes in phloem sap composition greatly differ from each other.

## 5. Conclusions

The hemp aphid *P. cannabis* exhibited mild stress in response to abiotic factors, including raised temperatures and herbicide application, resulting in increased fecundity. Hemp plants and hemp aphids are non-target species of the herbicide used in this study, which is the possible explanation for this observation. We have attributed this effect to hormesis. Some authors consider this phenomenon an important part of the adaptive response of organisms to fluctuations in environmental conditions [76]. Such fluctuations may precondition organisms to better cope with subsequent exposure to more severe stresses [75]. Hormesis could accelerate the growth of the pest population [80]. In our study, this mild stress resulted in a positive reproductive output, demonstrating the high ecological plasticity of the hemp aphid. The observed relationships were statistically significant; therefore, we believe it is worthwhile to undertake further studies on the composition of hemp plants, especially the concentration of plant protection product residues in the phloem sap, to better understand the observed insect behavior. Insight into the plant metabolome may help to predict the possible consequences of the observed phenomenon in the ecological, evolutionary and agronomic context.

## Figures and Tables

**Table 1:** Mean developmental time, longevity, and fecundity of *Phorodon cannabis* at different temperature regimes (*n* = 50). Values marked with different letters differ significantly at *p* < 0.05 based on the Tukey test.

Temperature	Pre-Reproduction	Reproduction	Post-Reproduction	Longevity	Fecundity
(°C)	Days	Days	Days	Days	Nymphs
	mean ± SD	mean ± SD	mean ± SD	mean ± SD	mean ± SD
20	11.0 ± 0.2 a	14.7 ± 11.1 a	0.0 ± 0.1 a	25.7 ± 11.2 a	54.5 ± 48.9 a
25	6.4 ± 0.5 b	15.2 ± 3.4 a	3.0 ± 2.6 b	24.6 ± 5.5 a	111.6 ± 17.7 b
28	6.0 ± 0 c	9.6 ± 3.3 b	2.6 ± 2.3 b	18.2 ± 5.1 b	27.8 ± 10.0 c
30	5.4 ± 0.5 d	2.1 ± 2.2 c	7.6 ± 4.9 c	15.1 ± 5.9 b	2.4 ± 2.9 d

**Table 2:** Life table parameters describing *Phorodon cannabis* as a function of temperature. Ro = net reproductive rate; rm = intrinsic rate of increase; λ = finite rate of increase; T = generation time; DT = doubling time

Temp. °C	rm	Ro	λ	T	DT
20	0.153	26.708	1.165	21.470	4.530
25	0.355	58.037	1.426	11.440	1.953
28	0.244	12.243	1.276	10.266	2.841
30	0.019	1.168	1.017	8.196	36.481

## Data Availability

Data supporting reported results are available on written request from the first author and the corresponding author.

## Supplementary Materials

The following are available online at https://www.mdpi.com/article/10.3390/insects12050420/s1: Table S1, The test of normality and analysis of variance.
