# Supplementary material for: Mild Abiotic Stress Affects Development and Stimulates Hormesis of Hemp Aphid Phorodon cannabis
Source: Insects. 2021 May 8;12(5):420. doi: 10.3390/insects12050420 (PMC8150316; doi:10.3390/insects12050420)
Supplement: Supplementary file 1 [file insects-12-00420-s001.zip › insects-1181495-supplementary.pdf]

# Supplementary Material: Mild Abiotic Stress Affects Development and Stimulates Hormesis of Hemp Aphid *Phorodon cannabis*

**Roma Durak, Malgorzata Jedryczka, Beata Czajka, Jan Dampc, Katarzyna Wielgusz and Beata Borowiak-Sobkowiak**

**Table S1.** The test of normality and analysis of variance.

| Variables | Normality | *p* - value | Analysis of Variance | *p*-Value |
|---|---|---|---|---|
| SOD | 0.953 [1] | 0.086 | 2.908 | 0.357 |
| CAT | 0.951 [1] | 0.073 | 3.157 | 0.571 |
| β-Glucosidase | 0.954 [1] | 0.093 | 2.471 | 0.996 |
| GST | 0.960 [1] | 0.151 | 6.756 | 0.818 |
| PPO | 0.959 [1] | 0.139 | 3.889 | 0.298 |
| POD | 0.954 [1] | 0.096 | 2.652 | 0.200 |
| aphid population (3 days) | 0.929 [1] | 0.295 | 0.637 | 0.648 |
| aphid population (5 days) | 0.881 [1] | 0.061 | 5.033 | 0.284 |
| aphid population (7 days) | 0.968 [1] | 0.851 | 1.868 | 0.193 |
| pre-reproduction | 0.075 [2] | 0.133 | 182.620 | 0.470 |
| reproduction | 0.146 [2] | 0.138 | 43.511 | 0.893 |
| post-reproduction | 0.293 [2] | 0.144 | 95.038 | 0.938 |
| longevity | 0.066 [2] | 0.151 | 15.013 | 0.069 |
| fecundity | 0.059 [2] | 0.129 | 51.911 | 0.351 |

Test of normality: [1] Shapiro-Wilk test, [2] Lilliefors test; Analysis of variance: Levene test.
